# Decoding complexity: tackling the challenge of how many transcription factors regulate a plant gene

**DOI:** 10.1080/21541264.2025.2521767

**Published:** 2025-06-25

**Authors:** John Gray, Erich Grotewold

**Affiliations:** a Department of Molecular, Cellular and Developmental Biology, University of Toledo, Toledo, OH, USA; b Department of Biochemistry and Molecular Biology, and Department of Plant Biology, Michigan State University, East Lansing, USA

**Keywords:** Transcriptional regulatory network, cis-regulatory element, protein-DNA interaction

## Abstract

The regulation of transcription is a major control point in the flow of information from the genome to the phenome. Central to this regulation are transcription factors (TFs), which bind specific DNA motifs in gene regulatory regions. In both metazoans and plants, 5–7% of all genes encode TFs. Although individual TFs can recognize and regulate thousands of target genes, an important question remains: how many TFs are required to precisely control the expression of a single gene? In this review, we compare the regulation of gene expression in plants and metazoans, outline key methodologies for identifying genes recognized or regulated by TFs, and explore what is currently known about the number of TFs needed to define the expression of any given plant gene. As the volume of high-throughput sequencing data continues to grow exponentially, it becomes increasingly clear that transcriptional regulatory networks exhibit remarkable complexity, characterized by many targets influenced by each TF; and that many TFs, often several dozens, contribute to the regulation of individual genes.

## Introduction

Controlling transcription is one of the most critical steps in determining how genetic information is expressed as phenotypes in any organism. In eukaryotes, the responsibility for transcription regulation largely hinges on a large group of proteins, the transcription factors (TFs) that recognize specific DNA motifs (known as *cis*-regulatory elements, CREs) in gene regulatory regions. Genes encoding TFs correspond to 5–7% of all genes in most eukaryotic genomes, and can be classified into distinct families based on defining features in their DNA-binding and/or protein–protein interaction domains [1–3]. Most eukaryotic TFs recognize CREs that are 6–10 nt long [4] arranged in *cis*-regulatory modules (CRMs). These CRMs are functional components of promoters, transcriptional enhancers, silencers, and insulator elements that ultimately determine in which cell(s), when, and at what level a particular gene is expressed [5]. Transcription start sites (TSSs) define the boundary between the transcribed region and what is normally known as the upstream gene regulatory region. Flanking the TSS, 50–100 bp to either side, is the core or basal promoter which serves as the assembly point for the pre-initiation complex, including RNA polymerase II (RNPII) and general transcription factors. The core promoter is often referred to as the “gateway to transcription”, because of its fundamental role in the integration of signals provided by TFs bound to regulatory regions and RNPII through the Mediator complex. The core promoter on its own only drives basal (low) levels of transcription [6,7]. Contrary to translation start sites, TSSs need to be empirically determined. In both plants and metazoans, there is significant variability in TSS selection [8,9], and because each alternate promoter is likely associated with its own regulatory elements (both proximal, but likely distal as well), TSS diversity adds a significant level of complexity to gene regulation.

**Figure 1.**
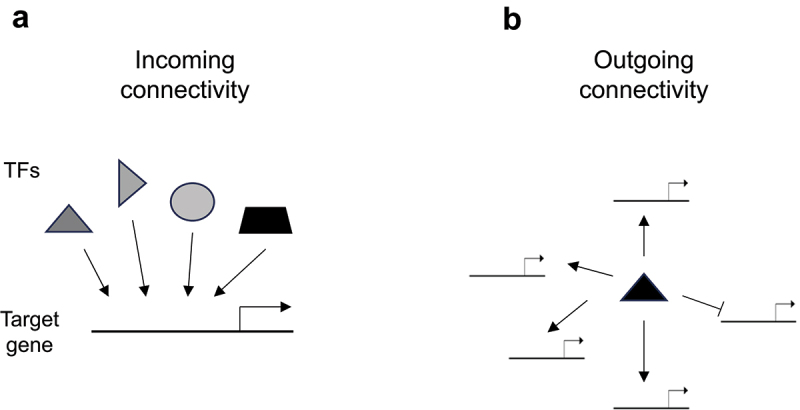
Diagram illustrating (a) incoming and (b) outgoing connectivity in transcriptional regulatory networks (TRNs). Target genes are indicated with a line and the transcription start site (TSS) with an arrow pointing to the right. Transcriptions factors (TFs) are indicated with the various solid shapes. Arrows from TFs to target genes indicate activation while a line ending in a perpendicular short line indicates repression.

TFs function in hierarchical arrangements, in which one TF often controls the expression of the gene encoding another TF (or itself) resulting in a complex system of interactions that are commonly known as transcriptional regulatory networks (TRN), which are similar to gene regulatory networks (GRNs) but restricted to transcription, without including other layers of gene regulation such as post-transcriptional, translational and post-translational processes. Moreover, GRNs include other components besides TFs and TF-target gene interactions such as chromatin accessibility and modifications, and RNA splicing. TRNs are characterized by the presence of recurring regulation patterns called network motifs. Such network motifs include coherent and incoherent feed-forward loops, single and multiple-input modules, dense overlapping regulons, bi-fan, autoregulation, and feed-through loop motifs [10]. TRNs are often represented as directed graphs, where edges signify regulatory interactions with a defined polarity. This polarity reflects the fact that a TF can bind to the regulatory region of a target gene, potentially encoding another TF, and modulate its expression, but the reverse interaction does not occur, as would be the case in a protein–protein interaction network. TRNs can be visualized from two complementary perspectives: the incoming connectivity, which considers how many TFs bind to the regulatory region of a particular gene; and the outgoing connectivity, which examines how many regulatory regions a single TF interacts with (Figure 1). TRNs globally display a scale-free architecture [11,12] meaning that most genes (nodes) have proportionally few interactions (a low degree), while a small number of genes are highly connected (the hubs). Unexpectedly, however, the exponents that characterize the power law that describe the distribution of the outgoing connectivity were found to be different across organisms suggesting that TF binding landscapes might have intrinsic organismal components, with the *C. elegans* out-degrees corresponding to a more “egalitarian” distribution (higher exponent) while *Arabidopsis thaliana* (*Arabidopsis*) showing a more “capitalistic” (lower exponent) behavior [13].

The analysis of genome sequences from thousands of organisms, combined with the advent of genome-wide tools to analyze mRNA accumulation and protein–DNA interactions both *in vivo* and *in vitro*, has unveiled an astonishing complexity in gene expression regulation that was unimaginable just a few decades ago. This review explores one particularly intriguing aspect of gene regulation that has seen significant advancements in recent years: the question of how many TFs are required to control the expression of any given gene. To start addressing this question, we first describe how TF-target interactions are identified, examine what is currently understood about their influence on gene transcription, and discuss the limitations of the various methods used to study these interactions. While the primary emphasis is on insights from plant systems, we draw comparisons with findings from metazoans where appropriate, offering a broader perspective. Rather than attempting to cover every discovery in this rapidly evolving field, the review narrows its focus to examine the critical issue of whether we are approaching a stage where it becomes feasible to determine and/or predict how many TFs are required to regulate any given (plant) gene.

## Plants and metazoans regulate gene expression similarly, with a few notable exceptions

While this article focuses on plants, it is essential to acknowledge that significant research on gene regulation has been conducted in metazoans. Comparing and contrasting the known differences and similarities between plant and metazoan gene regulation will provide valuable context and a broader understanding of the underlying mechanisms.

One of the most remarkable differences between the plant and metazoan transcriptional machinery is that, in addition to the DNA-dependent RNA polymerases I, II and III (RNPI, RNPII and RNPIII), respectively, responsible for transcribing ribosomal RNAs (rRNAs), messenger RNAs (mRNAs) and transfer RNAs (tRNAs), plants have two additional RNPs, RNPIV and RNPV identified for their participation in transcriptional silencing [14,15]. The main function of RNPIV is to generate 24 nucleotide-long small interfering RNAs (siRNAs) primarily from repeated DNA and transposable elements (TE), while RNPV produces long noncoding RNAs (lncRNAs) that participate in siRNA-mediated DNA methylation [16,17]. Indeed, RNA-directed DNA methylation (RdDM) is a major plant-specific epigenetic mechanism in the control of gene expression that involves RNPIV and RNPV [18]. RNPIV and RNPV are derived from RNPII, and while they have some subunits with distinct characteristics, the predicted subunit organization is largely conserved [19].

Since the previous paragraph centered on RNPs, central components of the basal transcriptional machinery, it is essential to highlight the Mediator complex. This complex serves a pivotal role in conveying regulatory signals from TFs to the transcriptional machinery anchored at the core promoter. Mediator links enhancers and target promoters through enhancer-promoter looping and regulates the chromatin architecture, collectively modulating the activity of RNPII. The Mediator complex is formed by 25–30 subunits organized into three core modules known as the head, middle, and tail, and a dissociable CDK module [20–23]. The subunit composition of the plant Mediator complex has been largely deduced from studies done initially in *Arabidopsis* [24], but subsequently expanded from studies in other plants [reviewed in [25]]. While interactions of a few TFs with specific plant Mediator subunits have been identified [26–34], a comprehensive Mediator-TF interaction map is still missing. Given the role of the Mediator complex in conveying signals from TFs bound to enhancer CREs to the transcriptional machinery, a comparison of plant and metazoans enhancers follows.

Plant and metazoans enhancers share many common characteristics, including their ability to function independently of orientation or location with respect to the transcribed unit, and are active over very long distances [35]. However, the histone modifications that characterize animal enhancers are absent in plant enhancers, and plant enhancers do not appear to produce the short-lived bidirectional enhancer RNAs that characterize animal enhancers [5,36].

The proportion of the protein-coding potential within genomes allocated to TFs is remarkably similar between plants and metazoans, ranging from 5% to 7%. Furthermore, many TF families, such as the basic helix-loop-helix (bHLH) and homeodomain (HD) families, are conserved across both kingdoms. However, certain TF families exhibit kingdom-specific specialization. For instance, WRKY TFs are unique to plants, while nuclear receptors are exclusively found in animals [37]. But even among those TF families that are conserved, there are lineage-specific characteristics that distinguish plants and metazoans. For example, MYB TFs represent a heterogeneous group of proteins that is ubiquitous in eukaryotes and expanded most notably in plants. MYBs are classified according to the number of repeats that conform the MYB domains. Vertebrate MYBs consist of three imperfect repeats (R1, R2, and R3), and 3 R-MYB proteins are also found in the plants [38], where they form a small gene family involved in cell cycle progression [39,40]. But most plant MYBs corresponds to the R2R3-MYB family, characterized by the presence of R2 and R3 MYB repeats. Each MYB repeat is formed by three α-helices, with the third α-helix making contacts with DNA [41]. The R2R3-MYB family is large, with ~ 130 members in *Arabidopsis* [42,43] and about twice that number in maize [44,45]. R2R3-MYB genes were proposed to have derived from an ancestral 3 R-MYB precursor by the loss of R1 [46]. Similarly, despite being present in all eukaryotes, plant and metazoans bHLH TFs have noteworthy differences. Animal bHLH proteins control processes such as neurogenesis, myogenesis, cell proliferation, muscle and bone formation [47–53]. Plant bHLH TFs control many cellular functions including stomatal formation and patterning, leaf hair (trichome) formation, light and hormone responses, and anthocyanin pigment formation [54–80]. bHLH domains consist of a basic (*b*) region that contacts the E-box DNA motif (CANNTG), and the HLH, mediating homo- and hetero-dimerization. HLH factors lacking the basic region can form homo- or heterodimers, but can’t bind DNA, hence function as DNA-binding inhibitors of their bHLH partners [55,70,73,81]. In addition, bHLH TFs often contain additional protein-protein interaction domains. For example, members of the MYC family of mammalian cell proliferation regulators (*e.g*., MAD, MNT), contain a leucine-zipper (LZ) that contributes to the selective interaction with MAX, another bHLH-LZ [82]. MAX forms homo- or heterodimers with several related proteins, such as MAD [83] and MNT [84]. MYC-MAX and MAX-MAX complexes bind E-boxes, yet only the MYC-MAX heterodimer activates cell proliferation genes. In contrast, MAX-MAD and MAX-MNT recruit histone deacetylase complexes resulting in transcriptional repression [85]. In contrast, the cooperation between a subset of R2R3-MYB and bHLH factors involving a solvent exposed surface in the MYB domain and a conserved MYB-interaction region (MIR) in the N-terminus of some bHLH provides one of the best described examples of combinatorial gene regulation in plants [86–88]. While the MIR is found in a rather small fraction of plant bHLHs, more than 30% of all plant bHLHs (the second largest TF family in plants with ~ 120 members present in *Arabidopsis*) harbor short (~85 amino acids) ACT-like (ACTL) domains that have been implicated in mediating bHLH TF homo- and hetero-dimerization, and which can modulate the DNA-binding activity of the bHLH [89–91]. These examples highlight how control of gene expression involves the coordinated action of multiple TFs in what is often known as combinatorial gene regulation [86], allowing a relatively small number of TFs to regulate a large set of genes with exquisite temporal and spatial expression patterns. Yet, to fully appreciate to what extent TF-binding sites are well known, it is important that we take a careful look at the methods by which TF occupancy is evaluated, and the limitations of each of the methods.

## Strengths and limitations of methods to determine protein–DNA interactions

The fact that protein–DNA interactions (PDIs) are highly dynamic presents a key challenge to assessing the binding of any given TF to a target CRE. A variety of techniques have been developed that can capture both stable and transient PDIs on a single gene or genome-wide scale and can be divided into TF-centered and gene-centered approaches [92]. Each technique has strengths and limitations that need to be considered when considering the identified PDIs for developing GRNs (Table 1).

**Table 1.** Advantages and disadvantages of methods for detecting PDIs.

	Advantages		Disadvantages/Limitations		Reference(s)
TF-centric approaches for PDI identification					
*ChIP-seq*	*In vivo* (plants or protoplasts) can detect PDIs in most plant tissues genome wide detection GFP-tagged TF may circumvent need for specific antisera		Requires specific antisera averages peaks across many cell types less favorable for capturing weak PDIs GFP-tagged TF requires transient/transgenic expression		[93,94]
*CUT&RUN*	*In vivo* (plants or protoplasts) can detect PDIs in most plant tissues genome wide detection needs 3 to 10X less cells than Chip-seq smaller fragments than Chip-seq and less background		requires specific antisera averages peaks across many cell types		[100,101]
*CUT&TAG*	*In vivo* (plants or protoplasts) can detect PDIs in most plant tissues genome wide detection preference for open chromatin regions		similar advantages over Chip-seq as CUT&RUN open chromatin preference may cause bias against heterochromatin regions		[103,104]
*TARGET*	*in vivo* (protoplasts) can identify low abundance TFs		isolated cells may miss complexity of *in planta* methods difficult to isolate protoplasts from some tissue types		[105,106]
*DAM-ID & TaDa*	*in vivo (*transgenics) Can identify transient PDIs Targeted DAM-ID (TaDA) involves inducible cell specific expression – requires much fewer cells		requires generation of transgenics bias toward GATC methylation sites spatial resolution is lower than Chip-seq		[110–112]
*PLA*	*in vivo* (any tissue) *in situ* technique permits single cell resolution		detecting PDIs requires specific antisera recognizing TF and dsDNA Has not yet been demonstrated in plants		[116–118]
*EMSA*	*in vitro* long established technique can identify PDI target down to single nucleotide resolution		*In vitro* conditions need optimizing and can greatly affect experimental outcome		[121,122]
*PBMs*	*in vitro* Can detect PDIs as well as small molecule interactions unbiased survey of DNA binding sites Halo-tag PBM uses cell free expression system		requires large amounts of protein and can be expensive, needs specialized equipment limited to detecting PDIs with high affinity limited to short 10-mer oligomer targets		[123–126]
*SELEX & SELEX-seq*	*in vitro* unbiased discovery of DNA target sequences needs lower amounts of protein than PBMs characterize TFs with/without post-translational modification can find DNA targets longer than 10-mers SELEX-seq more comprehensive than traditional SELEX cloning		may need to screen large number of clones *In vitro* conditions need optimizing and can greatly affect experimental outcome		[128,129,131]
*DAP-seq*	*in vitro* utilizes native genomic DNA (methylated or not) can compare TF occupancy in methylated vs non-methylated DNA		*In vitro* conditions need optimizing and can greatly affect experimental outcome does not capture the effects of chromatin accessibility, histone modifications, or other tissue-specific features that can influence TF binding		[211]
Gene-centric approaches for PDI identification					
*Y1H*	*in vivo* (using yeast as proxy eukaryotic cell) suitable environment to minimize TF folding issues finds PDIs irrespective of the TF being an activator or repressor overall is a reliable method for PDI discovery		requires large cDNA library or TFome may miss PDIs dependent on post-translational modification that does not occur in yeast will miss some PDIs if TF is not present in library still requires PDI validation by another method may miss heterodimeric TFs		[151–153,159]

### TF-centered approaches for PDI identification

#### ChIP-seq (Chromatin Immunoprecipitation sequencing)

The advent of high throughput sequencing enabled the genome wide readout of sites occupied by individual TFs that could be detected using antibodies. ChIP makes use of reversible cross-links between DNA and associated proteins by formaldehyde (or other cross-linker) fixation of cells or tissue [93]. The fixed chromatin is physically sheared and DNA fragments associated with a particular protein are selectively immunoprecipitated by specific antisera and analyzed. The bound DNA fragments are identified by PCR (ChIP-PCR), hybridization to a DNA microarray (ChIP–chip), or more likely today, by high throughput sequencing and mapped to a reference genome (ChIP-seq) [94]. This generates a global picture of where the TF binds, in contrast to simply interrogating single-binding sites by PCR (ChIP-PCR) [95]. The ChIP-seq derived data results in DNA segments (~200–1,000 bp long) containing the functional TF-binding sites, which can be identified by motif discovery or motif enrichment algorithms such as MEME [96,97] or FIMO [98]. ChIP-seq has been widely adopted but is limited in practice by the access to antisera that will precipitate specific TFs and their close relatives. This limitation can be circumvented if a TF tagged (*i.e*. GFP) fusion protein can be transiently expressed in protoplasts or transgenic plants and if the tag does not interfere with the function of the TF. ChIP-seq preferentially detects stable PDIs and is less favorable in capturing weak or transient (“touch and go”) PDIs [99]. ChIP-seq and its variants (such as CUT&RUN, see below) are now considered the gold standards to demonstrate *in vivo* PDIs.

#### CUT&RUN (Cleavage Under Targets & Release Using Nuclease) and CUT&TAG (Cleavage Under Targets & Tagmentation)

CUT&RUN is an alternative but analogous method to ChIP-seq for *in situ* genome-wide profiling [100–102]. ChIP-seq requires sonication to fragment the chromatin. In contrast, CUT&RUN employs protein A and protein G (pAG) to “tether” a micrococcal nuclease (MNase) enzymatic domain to antibody-bound chromatin in immobilized cells (or nuclei) for controlled and site-selective cleavage *in situ*. Controlled cleavage by MNase releases specific protein-DNA complexes into the supernatant for paired-end DNA sequencing, leaving the vast majority of DNA behind. The procedure tends to produce smaller DNA fragments than ChIP-seq with less background. This translates into requiring shallower (3 to 10-fold less) sequencing depth and a cleaner, sharper enrichment profile at target sites. As a result CUT&RUN has a higher resolution and reduced signal-to-noise ratio [101]. CUT&RUN does not necessitate the isolation of nuclei and as little as 1,000 cells can yield high-quality data for a TF. The reduction in cell inputs and sequencing depths lead to reduced costs permitting researchers to increase the number of experimental conditions that are tested. CUT&TAG is a derivative method whereby the pAG is fused to a hyperactive *Tn5* transposase loaded with sequencing adaptors (pAG-Tn5). This targeting of *Tn5* contrasts with the lack of targeting of *Tn5* tagmentation in the assay for transposase-accessible chromatin with sequencing (ATAC-seq) that maps nucleosome-depleted accessible chromatin. The *Tn5* transposase used in CUT&Tag prefers open chromatin regions, where transcription is more active but may introduce some bias against heterochromatic regions [103,104].

#### TARGET (Transient Assay Reporting Genome-wide Effects of Transcription Factors)

It provides a plant cell-based assay used to identify direct TF target gene interactions with timed nuclear entry of the TF [105,106]. Before being called TARGET, this technique was extensively used to identify targets for plant TFs in *Arabidopsis* [107–109]. The TF is introduced often as a fusion protein with a GR (glucocorticoid receptor) domain, in intact plants or isolated plant cells (protoplasts). The TF-GR fusion protein allows for temporal control of TF activity through sequential treatment with cycloheximide (CHX) and dexamethasone (DEX). CHX blocks translation of downstream regulators, and DEX induces TF-GR nuclear entry, allowing researchers to assess the direct targets of the TF. TARGET is a relatively fast assay compared to methods requiring stable transgenics or mutants, and it can be scaled up for higher throughput. It focuses on identifying direct targets of the TF, based on changes in gene expression, without relying on DNA binding information. It is broadly applicable as the use of isolated cells allows for the study of TFs in various tissues, if cell-based transient expression systems are available. However, by performing the assay in isolated cells, the complex biological interactions and feedback loops that occur in the whole plant may not be captured. The large datasets generated by TARGET require careful computational analysis to distinguish direct from indirect targets.

#### DAM-ID (DNA Adenine Methyltransferase Identification)

This method uses DNA methylation of promoters to detect highly transient TF-DNA binding interactions [110–112]. The Dam enzyme methylates adenines at GATC sites in DNA. When Dam is fused to a TF of interest, the fusion protein will methylate the DNA near its binding sites. This methylated DNA fragments are enriched, sequenced and used to identify the protein’s genomic targets. DamID can identify interactions that occur transiently because the Dam-mediated methylation persists even after the fusion protein dissociates. Since DamID does not rely on antibodies, it is suitable for studying proteins where antibodies are not available or are not highly specific. A derivation of this method, named *Ta*rgeted *DA*M-ID (TaDa), involves inducible cell type-specific expression to identify genome-wide protein/DNA interactions in 100 to 1000 times fewer cells than ChIP-based approaches, and has been applied to mammalian systems (mammalian TaDa or MaTaDa) [113].

There are significant limitations of this method (Table 1). For example, it requires generating transgenic organisms, which is not always easy or possible. In addition, the TF-Dam fusion protein may not function like the native TF, thus diminishing or eliminating the ability to interpret the data in a meaningful manner. Another limitation of this technique is that DamID relies on GATC motifs for methylation, which can lead to a bias toward regions with high GATC density. Also, the spatial resolution of DamID is generally lower than that of ChIP, making it less precise in identifying the exact location of protein binding [114].

#### PLA (Proximity Ligation Assay)

PLA was first developed as a highly sensitive method for detecting protein–protein interactions *in situ* [115]. However, this method was adapted to detect PDIs *in situ* by employing an anti‐double‐stranded DNA (dsDNA) antibody in conjunction with an anti‐TF antibody [116–118]. When the antibody recognizing the TF is in proximity (<40 nm) to the anti-dsDNA antibody, then DNA oligonucleotides attached to the antibodies hybridize and are ligated, forming a circular DNA template. This template is then amplified by rolling circle amplification, resulting in a fluorescent signal that can be visualized under a confocal microscope. This method was successfully applied to detecting the *in situ* binding of HSF1 to DNA in animal cells [116,118]. To our knowledge, however, this technique has not yet been applied to plant cells. It has the advantage of being able to detect TF binding at single-cell resolution in contrast to methods like ChIP-seq which can only provide information on the population mean of many cells. Like ChIP, PLA requires specific antibodies that often are not available and thus has not yet been widely employed. Different from ChIP and other TF-centered approaches, PLA does not provide definition for the specific DNA sequence that a TF binds. PLA has been used, however, to successfully study plant TF-protein interactions *in vivo* and has been shown to be suitable for detecting partners of low abundance TFs such as the FAMA TF in guard cells [119,120].

#### EMSA (Electrophoretic Mobility Shift Assay)

EMSA is a long established method used to study protein–DNA interactions qualitatively and quantitatively by observing the change in the mobility of dsDNA fragments when they bind to a protein in a gel electrophoresis system [121,122]. Since protein-DNA complexes are larger than free DNA, they migrate slower through the gel, resulting in a shift in the DNA band position. By using synthetic DNA targets, this method is particularly useful for identifying the specific DNA sequence motifs that TFs bind to with single-base-pair resolution. However, since this is an *in vitro* technique, factors like protein concentration, salt concentration, and gel conditions can affect the outcomes so careful optimization of experimental conditions to ensure accurate results [121].

#### Protein Binding Microarrays (PBMs)

Most of the methods developed for studying TF-DNA interactions can only be used to determine DNA-binding specificities of individual TFs. The development of TF-PBMs permits the screening for both biochemical activities and protein-protein/DNA/small molecule interaction efficiently for many TFs. The first TF-PBM developed for plants included 802 TFs derived from the *Arabidopsis* TF open reading frame (TFome) collection [123,124]. That microarray was employed to define the DNA-binding profiles of AP2/ERF family proteins in *Arabidopsis*. It also revealed four TFs (MYB-like, TCP and TLP TFs) that bound the EE (evening element) and had the expected phased gene expression under clock-regulation. In addition, the PBM was used to detect protein interactions including novel partners that interact with HY5 [123].

While PBMs can provide an unbiased survey of DNA-binding sites the method has several limitations (Table 1). The method studies PDIs *in vitro* and requires relatively high amounts of purified proteins, which can be challenging to prepare. It is limited to the detection of PDIs with high affinity because of extensive washing and at best it provides a semi-quantitative measurement of DNA binding as it is limited to short oligomers up to 10-mer [125]. Overall developing and using protein microarrays can be expensive and requires specialized equipment and expertise. For these reasons, PBMs have not been developed in other plant model systems to our knowledge. A 12,000 ORF Halo-tag PBM from *Arabidopsis* was later developed using a cell-free expression system and used to discover many novel TF-protein interactions, several of which could be validated using bimolecular fluorescence complementation (BiFC) assay [126].

#### SELEX (Systematic Evolution of Ligands by Exponential Enrichment)

SELEX is a technique used to identify high-affinity nucleic acid ligands (aptamers) that bind to a specific TF [127]. It involves iterative rounds of selection and amplification of a library of random DNA or RNA sequences, enriching for those that bind to the target, and then cloning and sequencing them [128]. SELEX-seq is a derivative method that employs high throughput sequencing at each cycle of SELEX allowing the determination of the relative affinities of any DNA sequence for any TF or TF complex [129]. The advantages of SELEX are that it requires low amounts of purified TF and one can characterize full-length TFs or TFs requiring posttranslational modification [125,130]. Unlike PBMs, SELEX can be used to identify target sequences more than 10 base pairs in length. Like PBMs it has the disadvantage of being *in vitro* and large pools of clones must be screened. In more recent years the *in vitro* method called DAP-seq (below) has gained favor compared to SELEX as it employs genomic DNA from the species of interest instead of random aptamers. However, both SELEX and DAP-seq (DNA Affinity Purification sequencing) can provide complementary information. For example, in a recent study, *Arabidopsis* WUSCHEL-related homeobox14 (AtWOX14) was investigated using both techniques and in the presence or absence of cytosine methylation. It was found that ^5m^C represses the DNA binding of AtWOX14 monomers but facilitates the binding of its dimers, and the methylation effect on a cytosine’s affinity to AtWOX14 is position dependent. This methylation change has the potential to rewire the regulatory network downstream of AtWOX14 uncovering an unrecognized effect of DNA methylation on the binding of this TF [131].

#### DAP-seq (DNA Affinity Purification sequencing

Provides a high-throughput TF-DNA binding discovery assay that uses genomic DNA and TFs expressed *in vitro*. DNA libraries are constructed using native genomic DNA, thus preserving DNA methylation marks that can affect TF binding, unless the library is PCR-amplified, in which case methylation is lost, allowing to compare DNA-binding preferences for methylated and unmethylated DNA. The DNA library is incubated with an affinity-tagged *in vitro*-expressed TF, and TF-DNA complexes are purified using magnetic separation of the affinity tag. Bound genomic DNA is eluted from the TF and sequenced using next-generation sequencing. Sequence reads are mapped to a reference genome, identifying genome-wide binding locations for each TF assayed, from which sequence motifs can then be derived [132,133]. Since DAP-seq uses genomic DNA in a chromatin-free context, it does not capture the effects of chromatin accessibility, histone modifications, or other tissue-specific features that can influence TF binding *in vivo*. To address this, DAP-seq data can be integrated with other methods like DNase-seq, ATAC-seq, or MNase-seq. Since DAP-seq is performed *in vitro*, the buffer conditions can affect protein folding and complex formation, potentially impacting TF functionality and binding behavior. Another limitation of DAP-seq is that certain TFs either require heterodimerization to bind DNA or have altered DNA binding properties when combined with other TFs [134,135]. This can be tackled using double DAP-seq (dDAP-seq) whereby two TFs are tagged separately (*e.g.*, HALO and SBP) and the peaks unique to where the two proteins are present are inferred to represent binding enabled by heterodimerization. Using dDAP-seq, it has been shown for 20 bZIP TF pairs from *Arabidopsis* that heterodimers show distinct preferences for the ACGT elements recognized by plant bZIPs [136]. Similarly, using sequential DAPseq (seqDAP-seq) in combination with ChIP-seq and expression data, it was shown that tetrameric complexes of the MADS box TFs SEPALLATA3 (SEP3) and AGAMOUS (AG) could access new sites and demonstrated a global increase in DNA-binding affinity compared to the TFs operating alone [137].

#### Perspectives on computational methods to assess PDIs

While experimental methods, such as ChIP-seq and Y1H assays, remain the gold standard for identifying TF binding to CREs, computational approaches have also been employed to predict CREs involved in functional PDIs. A key challenge in this endeavor is the high frequency with which short CRE motifs occur in the genome by chance, raising questions about their functional relevance in gene regulation. Studies in yeast, humans, and *Arabidopsis* have shown that CREs are not randomly distributed within promoters, but instead are enriched near TSSs [138–140]. Importantly, conservation of CRE position across related species provides further support for their regulatory significance. For instance, by applying a conservation filter across *Arabidopsis lyrata, Brassica oleracea*, and *Brassica rapa*, Yu *et al*. (2016) demonstrated that conserved CREs in Arabidopsis promoters exhibit a near bell-shaped distribution, peaking approximately 50 bp upstream of the TSS [141]. Additionally, some CREs preferentially localize to the first intron, as observed in *Arabidopsis* and other eukaryotes [142].

In recent years, machine learning (ML) approaches have been increasingly applied to decipher the relationship between non-coding regulatory sequences and gene expression. A cross-species ML study in Arabidopsis, tomato, sorghum, and maize identified a set of predictive CREs that could define expression-predictive motifs (EPMs), enabling accurate expression prediction among closely related species [143]. However, such models are less effective in capturing species-specific CREs. Another ML-based framework, Plant-DTI (Plant DNA-binding Domain – TF Binding Site Interaction), was developed to improve the mapping of TF to their preferred CREs [144]. This predictor successfully identified MeERF72 as a regulator of *MeSUS1* in cassava, a prediction subsequently supported by yeast one-hybrid (Y1H) validation.

Conserved CREs have also been identified through positional analysis of promoter regions, yielding the so-called preferentially located motifs (PLMs) [145]. Although many PLMs correspond to known TF or miRNA binding sites, a comparative analysis between *Arabidopsis* and maize revealed that up to 79% of the identified PLMs remain unassigned to known regulators [146]. As predictive tools continue to improve, particularly when integrated with large-scale datasets such as ChIP-seq, RNA-seq, and curated CRE databases, they are expected to play an increasingly central role in mapping PDIs and expanding our understanding of plant *cis*-regulatory vocabularies [147–150].

### Gene-centered approaches for PDI identification

#### Yeast One-Hybrid (Y1H)

The Y1H method aims to identify which TFs from a TFome bind to a particular DNA regulatory region (target). In Y1H, the expression construct includes a fusion protein between a TF of interest and the transcriptional activation domain (AD) of the yeast GAL4 protein. Since the fusion protein includes the yeast AD, irrespective of whether the TF is activator or repressor, the TF activates a reporter gene (bait construct) in a yeast host if it physically binds to it [151]. The bait strain is typically screened with an entire prey library for discovery or by individual prey constructs for detailed PDI studies [116,152,153]. Y1H screening is dependent on the availability of a comprehensive cDNA or TFome library and has been employed successfully in several model plant species, including *Arabidopsis*, tobacco, rice and maize [154–158]. By comparing the data from one of these screens with published PDI results, estimates of Y1H precision, were estimated to be ~ 0.90, recall ~ 0.73 and false positive rate ~ 0.092, indicating that Y1H provides a robust and reliable method for PDI discovery [155]. Although Y1H relies on PDIs occurring in a heterologous system, because it is *in vivo*, it provides a suitable environment for prey protein folding and secondary TF modifications, thus minimizing limitations of *in vitro* methods. The method allows for the identification of double positive transformants from a single yeast colony, which could indicate a requirement for protein dimerization. Disadvantages of Y1H include the fact that validation of candidate PDIs is still required in the actual organism from which the TFs are derived. Although yeast acts a proxy for any eukaryotic cell, PDIs (false negatives) might be missed if a TF requires a post translational modification to bind DNA that is not provided in the yeast cell or if incorrect protein folding occurs. The requirement of a complete or near complete TF or cDNA library for a specific organism limits the use of this technique to identify PDIs on a large scale. False negatives may occur if some TFs are missing from a screening library, and since this method does not reliably detect heterodimers. it will fail to detect TFs that require heterodimerization [152,159].

While each of the methods for discovering PDIs described above has some limitations, they are together responsible for the rapid advances that continue to be made in elucidating the complexities of gene regulation in plants and other eukaryotes. In addition, they require *in planta* validation, with traditional methods including ChIP-qPCR or dual luciferase assays, often conducted transiently in plant protoplasts or by *Agrobacterium*-mediated infiltration in *Nicotiana benthamiana* leaves [160–164].

## From focal TF to large TF complexes facilitated by intricate CRE arrangements

For decades, mutant analyses have been instrumental in uncovering phenotypes linked to the loss-of-function of individual TFs, often leading to striking phenotypic changes. Examples include classical patterning mutants in *Drosophila melanogaster* (fruit fly) that resulted in the discovery of HD TFs [165], flower homeotic mutations in *Arabidopsis* resulting from loss of MADS-box TF gene function that provided the earliest evidence for the molecular basis of floral organ specification [166], and pigment mutants in maize that contributed to the discovery of transposable elements [167] that were subsequently shown to encode R2R3-MYB and bHLH TFs [74,168]. These findings led the scientific community to widely adopt the assumption that one or a few TFs would be both necessary and likely sufficient to regulate the expression of a given gene. This perspective was consistently conveyed to students over many years, shaping their understanding of gene regulation. It is undeniable that the loss of a single TF can often cause significant changes in the expression of one or more the genes that it is associated with, frequently resulting in observable phenotypic outcomes. To distinguish these TFs from others involved in the gene’s regulation, whose impacts may be subtler or less readily apparent, we have referred to these critical TFs in the past as *focal transcription factors* [or fTFs [169]]. While fTF mutations can be very informative with regard to the role that the TF plays in the regulation of a gene set or process, it is a combination of many TFs that ultimately contribute to the appropriate expression of a gene in space, time and level.

Concomitantly, the CRE landscape in the vicinity of a target gene determines the repertoire of TFs that can participate in its regulation. The dissection of the mechanisms responsible for deploying the expression of the sea urchin *Endo16* gene in the endoderm during embryo development provides one of the earliest examples of the complexity of gene regulation. Within the 2.3 kb regulatory region upstream of the *Endo16* transcription start site (TSS), researchers identified at least six CRMs comprising a total of 36 CREs that interact with at least seven distinct TFs [170].

**Figure 2.**
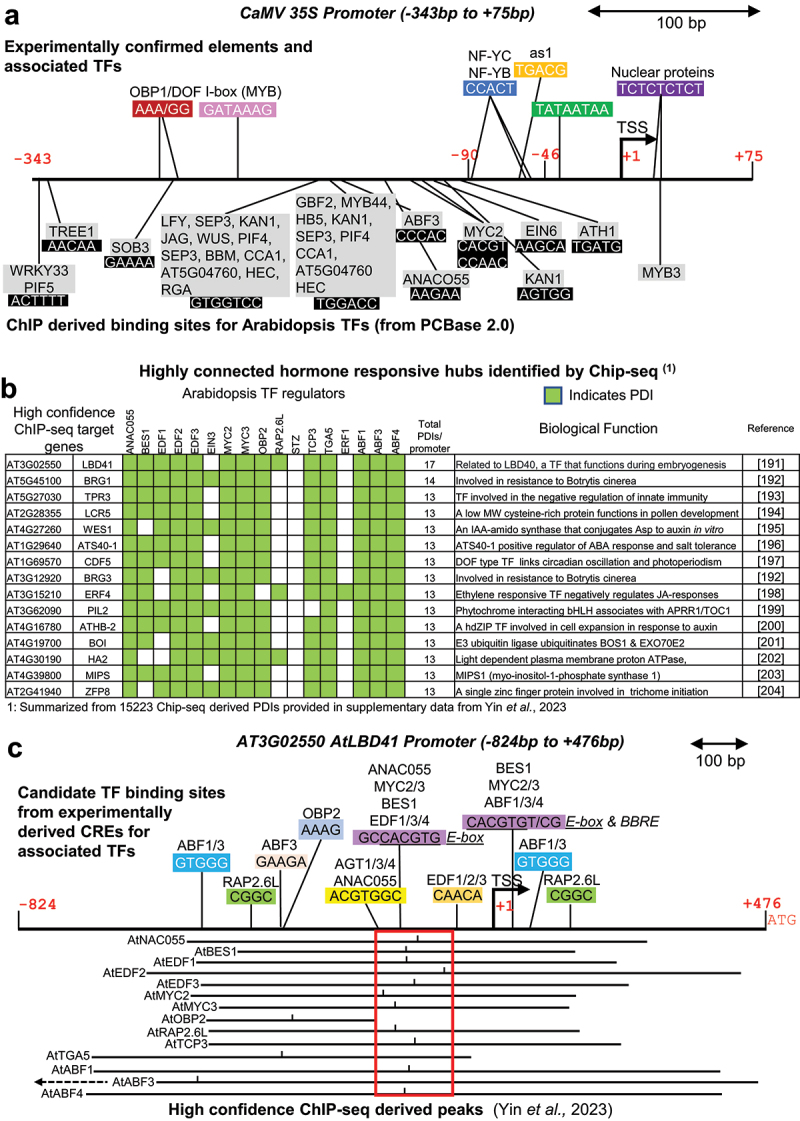
Examples of plant hub genes that exhibit high incoming connectivity. (a) *CaMV 35S* promoter. Scaled diagram summarizes experimentally derived CREs and associated TFs (colored boxes) located in the *CaMV 35S* promoter region (GenBank V00140.1). The TATA box is shown in green and three CAAT boxes in blue. The colored elements have been confirmed by one or more methods (see text for details). TSS corresponds to the transcriptional start site. The elements shown in black are derived from plant ChIP-seq database (PCBase 2.0) and associated TFs from *Arabidopsis thaliana* are shown by gray boxes. This database collects and utilizes plant ChIP-seq experimental data derived from gene expression omnibus (GEO) and sequence read archive (SRA) under various conditions to infer transcription binding sites for seven model plants. Only CREs with a similarity score of 1.0 are shown. (b) Table of highly connected hormone responsive hub genes identified by ChIP-seq in *Arabidopsis* . The list of most highly connected genes that are targeted by a set of 17 hormone responsive TFs is derived from supplementary data provided in yin *et al.*, 2023 [186]. PDIs supported by high confidence ChIP-seq peaks are indicated by green cells. (c): diagram of the *AtLBD41* (At3G02550) promoter. Scaled diagram summarizes the locations of high confidence ChIP-seq peaks that map to the promoter region of *AtLBD41*. The center of each peak is indicated by a tick mark and the red box highlights a tight clustering of CREs near these peak centers. The location of CREs that have been shown to bind each respective TF are shown above the line. CREs are derived from PCBase 2.0 and the plant promoter analysis navigator (PlantPan 4.0).

Viruses have evolved to contain strong promoters with multiple CREs, and the study of gene regulation in animal viruses has significantly shaped our understanding of eukaryotic gene control, offering key insights into promoter architecture and transcriptional regulation [reviewed in [171,172]]. This is also the case in plants, with the *Cauliflower Mosaic Virus* (*CaMV*) *35S* promoter standing out as one of the most well-studied examples of highly complex promoter architecture [173–176]. The *CaMV 35S* promoter has been extensively used as a powerful tool in plant biotechnology due to its ability to drive high levels of gene expression across a wide range of plant tissues and species, making it a cornerstone in the development of genetically engineered crops [177]. *CaMV 35S* has a modular architecture including a TATA box and three CAAT boxes in the minimal promoter region (Figure 2(a)). The 5’ UTR region contains a CT (inverted GA) repeat that is involved both in the enhancer function and in the interaction with plant nuclear proteins [178]. Within the minimal *CaMV 35S* promoter, the *as1* (TGACG) CRE was defined (Figure 2(a)) as recognized by the *Nicotiana tabacum* TGA1a and TGA1b bZIP TFs [174,179]. Further upstream in the *CaMV 35S* promoter, *Arabidopsis* OBP1 (AtOBP1, At3G50410) recognizes the 5”-AA[AG]G-3” motif [180]. AtOBP1 (a.k.a. DOF3.4) contains a DOF (DNA binding with one finger) domain that harbors a C2C2-type zinc finger motif that is expressed in meristems and has been implicated in regulating the cell cycle [181,182]. Several other TFs have been proposed to recognize conserved CREs in the *CaMV 35S* promoter, with some confirmed through ChIP-seq analysis (Figure 2(a)), while many others remain predicted but experimentally unverified.

An example of complex plant gene regulation is provided by GIGANTEA (*GI*). *GI* encodes a scaffolding protein that plays crucial roles in photoperiod-controlled flowering, circadian clock regulation, and photoreceptor signaling. *GI* transcription is tightly regulated by both the circadian clock and light [183]. Within a 3 kb region of the *Arabidopsis GI* promoter, three conserved CRMs have been identified across plant species. Among these, the CRM2 region was shown to drive rhythmic expression. CRM2 contains three ABA Response Element-Like (ABREL) motifs and three Evening Elements (EEs). The ABREL motifs are predicted to be recognized by bZIP TFs, which enhance gene expression in the evening. The EE elements, in contrast, are bound in the morning by LHY and CCA1, which suppress *GI* expression during this time. Later in the day, REV8 (MYB) binds near the same EE motifs to activate *GI* transcription [184].

In maize, ChIP-seq and yeast one-hybrid assays (Y1H, see below) identified nine and ten TFs, respectively, that recognized the *Zm4CL3* and *ZmCOMT1* promoters, showing that genes that encode enzymes in the phenylpropanoid pathway can exhibit high incoming connectivity [157]. A follow-up Y1H study investigated the layered nature of the TRN associated with the regulation of maize phenolic compound accumulation. This study found that 19 Tier Two TFs (Tier Two corresponds to TFs that directly control genes for biosynthesis enzymes) could transactivate 6–19 out of 18 predominantly expressed phenolic genes. In that study, the number of *ZmCOMT1* regulators was increased to 17 [154], with 15 of the Tier Two TFs in turn controlled, on average, by 10–16 Tier Three TFs (Tier Three TFs being defined as TFs that regulate genes encoding Tier Two TFs). Together, these studies revealed that for these predominantly expressed phenolic compound biosynthesis genes, there is complex regulation with high incoming connectivity for genes at different levels (tiers) in the regulatory hierarchy. One interpretation of this connectivity is that it is required for the fine tuning of phenolic compound production in a temporal and tissue-specific fashion.

A similarly high level of incoming connectivity was found for some genes in *Arabidopsis*. An earlier ChIP-seq study of 27 TFs constructed an experimental network containing 46,619 regulatory interactions and 15,188 target genes [185]. This has led to the identification of hub targets (≥7 TFs binding) which comprised ~11% of all the interactions. These hubs were enriched for genes involved in development, responses to various stimuli, signaling, and gene regulatory processes. A more recent ChIP-seq study conducted on 17 TFs known to mediate hormone responses in *Arabidopsis* identified 96,089 high confidence ChIP-seq peaks on 15,223 unique promoter targets [186]. Of these TF target genes, 2,029 (13%) had an incoming connectivity of ≥7 TFs and were described as hubs, while 25 targets had as many as 14 TF peaks that mapped to their promoter regions (Figure 2(b)). These highly connected hubs encode TFs and hormone-related genes linked to development and disease resistance. One of these highly connected genes corresponded to *AtLBD40* encoding a LOB domain TF expressed primarily in embryogenic and meristematic tissues. The ChIP-seq peaks for *AtLBD40* were located within a 110 bp region immediately upstream of the TSS (Figure 2(c)). Given that these studies were conducted with only 1–2% of all the TFs, they strongly suggest that ~10% of the plant genes display a very high incoming connectivity. Again, this high connectivity may be a requirement for the ability of sessile plants to fine tune growth in response to many signals during development and during exposure to biotic and abiotic stresses.

What characterizes the promoters of the genes that display high incoming connectivity is the presence of many CRMs, each with multiple CREs recognized by several TFs that ultimately contribute to gene regulation. In metazoans, it is not uncommon for the same TF to bind to multiple CREs within a CRM in what are known as homotypic clusters, often interspersed with binding sites for multiple TFs [187]. The presence or frequency of homotypic clusters has not been investigated as extensively in plants, as in metazoans.

## Evaluating TF-target gene binding as a proxy for gene regulation

Based on what is known today, the recruitment of a TF to a regulatory complex serves one function, which is to influence the rate or amplitude of transcription of the gene controlled by the regulatory complex. It is, however, common to find that a TF binds *in vivo* to the regulatory region of a gene without a noticeable change in mRNA accumulation [188]. Putting aside the technical challenges associated with ChIP-associated methods (Table 1) [189,190], the observed discordance between TF binding and gene expression can also be a consequence of how mRNA accumulation is measured. For example, RNPII pausing, a phenomenon that is found at a significant proportion of metazoan genes [191], results in the formation of a < 100 nucleotide-long nascent RNA that would be difficult to detect by conventional mRNA profiling since the nascent RNA is short and not polyadenylated. RNPII pausing in plants has different characteristics from metazoans [192], with initial studies suggesting rare promoter-proximal pausing but much more frequent pausing close to the polyadenylation site [193], while more recent studies using global run-on sequencing (GRO-seq) showed that RNA polymerase II pauses right after the TSS in ~20% of all *Arabidopsis* genes [194]. Another possible explanation for the observed discordance between TF-location studies (such as ChIP-seq) and transcriptional outputs is the reliance on steady-state mRNA levels (as determined by RNA-seq, for example) as a proxy for transcriptional activity [195]. This problem is particularly accentuated in the transient gene expression changes induced by stress, where mRNAs for transcriptionally induced genes are often destabilized, and mRNAs for repressed genes are often more stable [196].

The emerging picture from these studies highlights the imprecision of current methods in evaluating the functional transcriptional output of a TF bound to a gene’s regulatory region. Compounding this challenge is the limited understanding of what defines a functionally relevant TF-target interaction. The interpretation of ChIP-seq experiments, particularly decisions regarding signal thresholds in the context of the number of replicates and controls used, can significantly influence the selection of TF targets, leading to the identification of interactions across a broad spectrum of binding affinities. If low levels of DNA occupancy in ChIP-seq experiments are interpreted as evidence of lower affinity between a TF and its binding sites, it may lead to the assumption that such low-affinity binding sites are less significant for gene regulation, as posited in models that suggest that transcription networks are continuous, rather than discrete [188]. However, emerging evidence indicates that low-affinity TF binding sites play critical roles in fine-tuning gene expression, particularly in combinatorial and context-dependent regulatory mechanisms [197–199]. Such low-affinity binding sites are usually not captured by position weight matrices (PWMs), which are mathematical representations of the likelihood of each nucleotide occurring at every position within a specific DNA sequence motif, and are frequently used to describe TF binding preferences [200]. Thus, while powerful in describing the *in vitro* binding properties of TFs, PWMs provide poor predictors of CRE functionality *in vivo* [197].

Returning to one of the central questions, this study seeks to address – how many TFs are required to regulate a single plant gene – the answer depends heavily on how TF-target interactions are defined and counted. Should the focus be limited to highly reproducible targets identified through ChIP-seq experiments, or should the analysis also include potential targets that exhibit corresponding changes in mRNA accumulation? The criteria chosen will significantly influence the interpretation and complexity of gene regulation.

Recently, this question was addressed in a comprehensive manner in yeast where the binding locations for nearly all yeast sequence-specific TFs was examined [201]. Each yeast TF was individually fused to micrococcal nuclease (MNase) and their positions on genomic DNA probed using chromatin endogenous cleavage with sequencing (ChEC-seq). It was found that 5,467 out of 5,891 promoters detectably interacted with at least one TF and a typical promoter interacts with a median of 15 TFs, but with a wide range of 1 to 137 TFs per promoter. It is interesting that only about one-third of the binding sites contained a binding motif so that for most TFs the presence or absence of a sequence motif cannot reliably predict TF – promoter binding *in vivo* [201].

## How many TFs are necessary to regulate a single (plant) gene?

This is a fundamental yet complex question, as the relationship between TF binding and regulatory output is not always linear. As noted earlier in this manuscript, TF binding does not necessarily result in functional regulation. Moreover, unless empirically determined, the regulatory space of any given gene is not established, with CREs potentially being hundreds of thousands of base pairs away, as is often the case with enhancers. While advances in single-cell transcriptomics and TF-binding assays have provided valuable insights, current single-cell methods are insufficient to determine combinatorial TF activity and direct regulatory outcomes. Thus, it remains unfeasible to fully address this question at single-cell resolution in plants or other organisms. Nonetheless, examples can be analyzed with the understanding that these studies integrate TF binding and expression outputs across spatial and temporal scales.

Inference of the *Arabidopsis* TRN based on the integration of available information on chromatin accessibility, TF-binding, differential gene expression, and expression-derived regulatory interactions involving ~1,500 TFs and ~31,400 target genes (1.7 M predicted interactions) suggested an average of 54 TF binding to any given gene. Indeed, about half of the genes in the inferred TRN were recognized by 8–79 TFs, and the genes with the highest incoming connectivity encoded other TFs [202]. About 2,200 genes only had one incoming TF and these genes encoded functions enriched in RNA binding and translation [202]. These numbers are very much in agreement with previous estimates suggesting on average ~75 TF binding events per *Arabidopsis* gene [203].

The *Arabidopsis* genome is compact, with one gene every ~5 kb [204], and ~1,770 TFs [205,206]. How are the numbers different when a much larger genome with a significant larger number of TFs considered? A large-scale ChIP-seq analysis in maize protoplasts using 104 TFs allowed the prediction of a network of ~272,000 edges and ~21,000 nodes (likely representing genes) [207]. Some quick back of the envelop calculations suggest that each of the 21,000 genes is recognized by an average of ~13 TFs. However, the maize genome encodes close to 2,500 TF [208]. Thus, if the 104 TFs selected in this study represent a random subset of all maize TFs, the average number of TFs recognizing any given gene is now closer to 310. In this regard, it is interesting that this study identified 76 distinct TFs (out of the 104 tested) that recognized a conserved noncoding sequence (CNS) previously identified as a major flowering-time quantitative trait locus, *Vegetative to generative transition 1* (*Vgt1*) located ~70 kb upstream of a flowering-time regulator [209]. More enhancer regions have been revealed in maize via the MaizeCode project, which integrates datasets that evaluate chromatin structure, with transcriptomic datasets to identify regions of the genome that could regulate and/or register gene expression [147,210]. These enhancer regions appear to be under rapid evolution during domestication from teosinte, however like the *Vgt1* locus the highest level of transcriptional conservation was observed in pollen-related enhancers likely because breeding relies on fecundity and genome stability [147].

## Conclusions and perspectives

It is evident that the last decades have provided a very significant advance to how we understand the control of transcription in eukaryotes. While plants and metazoans employ comparable mechanisms, the distinct differences between them shed light on the system’s inherent plasticity and reveal valuable opportunities for innovatively manipulating gene regulation. However, we are still far from being able to determine how any gene in a multi-cellular organism is regulated with exquisite temporal and spatial patterns. Despite extensive knowledge of TF-target gene interactions, the detailed information density of regulatory sequences, specifically, which base pairs are involved and what fraction contribute functionally, remains poorly understood for most genes. This gap highlights a critical need to map and quantify the precise contributions of individual base pairs to regulatory function, offering potential insights into the evolutionary and functions constrains of gene regulatory systems. It is evident that many TFs are required for the regulation of any given gene – how many, depends on the gene, but can range from 10’s to 100’s. Still, it is likely that only one or a few of such TFs (what we call the fTFs) have the most significant effect, resulting in obvious phenotypic defects. How all these TFs interact with each other and with Mediator and components of the basal transcription machinery to convey precise regulatory signals remains largely unknown.
